# Age-Related Changes in the Natural Killer Cell Response to Seasonal Influenza Vaccination Are Not Influenced by a Synbiotic: a Randomised Controlled Trial

**DOI:** 10.3389/fimmu.2018.00591

**Published:** 2018-03-22

**Authors:** Agnieszka Przemska-Kosicka, Caroline E. Childs, Catherine Maidens, Honglin Dong, Susan Todd, Margot A. Gosney, Kieran Michael Tuohy, Parveen Yaqoob

**Affiliations:** ^1^Department of Food and Nutritional Sciences, University of Reading, Reading, United Kingdom; ^2^Department of Mathematics and Statistics, University of Reading, Reading, United Kingdom; ^3^School of Psychology and Clinical Language Sciences (MAG), University of Reading, Reading, United Kingdom; ^4^Innovation Centre, Fondazione Edmund Mach, Trento, Italy

**Keywords:** aging, influenza, prebiotic, probiotic, vaccination

## Abstract

Natural killer (NK) cells are an important component of the immune response to influenza infection, but are subject to alteration during aging, which may play a role in impaired response to infection and vaccination in older people. Enhancement of NK cell activity could, therefore, present a means to improve the immune response to vaccination in older subjects, and pre- and probiotics offer an opportunity to modulate antiviral defenses *via* alteration of the gut microbiota. This study investigated the effect of a novel probiotic, *Bifidobacterium longum bv. infantis* CCUG 52486, combined with a prebiotic, gluco-oligosaccharide (*B. longum* + Gl-OS), on the NK cell response to seasonal influenza vaccination in young and older subjects in a double-blind, randomized controlled trial. There were significant effects of aging on NK cell phenotype, the most notable of which were an increase in CD56^dim^ cells, mainly reflected in the CD16^+^ subset, a decrease in CD56^bright^ cells, mainly reflected in the CD16^−^ subset, and greater expression of the immunosenescence marker, CD57, on NK cell subsets. However, these changes only partially translated to differences in NK cell activity, observed as trends toward reduced NK cell activity in older subjects when analyzed on a per cell basis. Influenza vaccination increased the proportion of CD56^bright^ cells and decreased the proportion of CD56^dim^ cells, in young, but not older subjects. Although NK cell activity in response to vaccination was not significantly different between the young and older subjects, low post-vaccination NK cell activity was associated with poor seroconversion in only the older subjects. There was no influence of the synbiotic on NK cell phenotype or activity, either before or after influenza vaccination. In conclusion, aging is associated with marked alteration of the phenotype of the NK cell population and there was evidence of an impaired NK cell response to influenza vaccination in older subjects. The effects of aging on NK cell phenotype and activity could not be offset by *B. longum* + Gl-OS.

**Clinical Trial Registration:**

www.ClinicalTrials.gov, identifier NCT01066377.

## Introduction

Influenza vaccination offers an important prophylactic solution for preventing infection and associated complications, but immunosenescence significantly impairs vaccine efficacy (1). Potential adjuvants and dietary strategies to improve the immune response to influenza vaccines in older people are, therefore, of great interest. Emerging evidence suggests that the resident gut microbiota plays an influential role in shaping antiviral defenses and modulating the outcome of viral infections (2), and these forms the basis for the hypothesis that pre- and probiotics may modulate responses to infection or vaccination.

Natural killer (NK) cells and NKT cells are an important component of the immune response to influenza infection and low NK activity may be related to higher infection risk (3). They are recruited to the respiratory tract 48 h after infection with influenza virus and interact with influenza virus hemagglutinin (HA) *via* receptors NKp44 and NKp46, leading to activation (4). Augmentation of NK cell activity after exposure to influenza virus-infected cells has been reported in both animals (5, 6) and humans (7), and NK cell-depleted mice have lower production of antibodies and inflammatory cytokines after mucosal immunization (8). Nevertheless, very little is known about the effect of influenza immunization on NK cell number or function, particularly in the context of aging, and it is not clear whether NK cells respond similarly to influenza vaccination in young vs older subjects (9, 10). The balance of evidence suggests that while total NK cell number increases during aging (11–14), there is a decline in NK cell activity on a per cell basis (15), a gradual accumulation of long-lived CD56^dim^ NK cells (16–18) and a decline in the CD56^bright^ subset in older subjects, which may lead to impaired cytokine production and adaptive immunity (17, 19–22). Enhancement of NK cell activity could, therefore, provide a means to improve the immune response to vaccination in older subjects.

Since aging is associated with reduced biodiversity and compromised stability of the gut microbiota (23), as well as immunosenescence, older individuals may derive benefit from intervention with pre- and/or probiotics. To date, studies examining the adjuvant effects of probiotics on the immune response to vaccination have focused exclusively on adaptive immunity, but this could be indirectly affected by NK cells. In support of this concept, administration of the probiotic, *Lactobacillus casei* Shirota, and *Lactobacillus rhamnosus*, protected mice from intranasally administered influenza virus infection at least partly by enhancing NK cell activation in the lung (24, 25). This paper examines the effects of a novel synbiotic on NK cell phenotype and activity before and after influenza vaccination in young and older subjects as secondary outcomes of the PRIMAGE (Probiotics, Immunity, and Aging) study (26).

## Materials and Methods

### Ethics and Trial Registration

The study protocol was reviewed and approved by the University of Reading Research Ethics Committee (project number: 10/09) and the National Health Service (NHS) Research Ethics Committee for Wales (10/MRE09/5). The trial was registered with clinicaltrials.gov (Identifier: NCT01066377) and conducted according to the guidelines laid down in the Declaration of Helsinki.

### Participants

Prior to the influenza season of 2010–2011, young (18–35 years) and older (60–85 years) healthy adults were recruited from the population in and around Reading (UK) through newspaper and poster advertisements, email, and radio. Inclusion criteria were: a signed consent form, age 18–35 years or 60–85 years, body mass index (BMI) 18.5–30 kg/m^2^, good general health, as determined by medical questionnaires and laboratory data from screening blood and urine sample (fasting glucose, erythrocyte sedimentation rate, full blood count, liver function tests, renal profile, dipstick urinalysis), not pregnant, lactating, or planning a pregnancy. Exclusion criteria included: allergy to the influenza vaccine, HIV infection, diabetes requiring any medication, asplenia, and other acquired or congenital immunodeficiencies, any autoimmune disease, including connective tissue diseases, malignancy, cirrhosis, current use of immunomodulating medication (including oral and inhaled steroids), self-reported symptoms of acute or recent infection (including use of antibiotics within past 3 months), taking lactulose or any other treatment for constipation, alcohol, and drug misuse. Additional exclusion criteria for older volunteers included: laboratory data which were outside the normal range for this age group AND outside the ranges specified in the SENIEUR protocol (27), Barthel Index score of <16/100, cumulative illness rating scale score of >15 (28). Additional exclusion criteria for the young subjects included laboratory data which were outside the normal range and influenza vaccination in the previous 12 months.

### Sample Size

The primary outcome of the trial was the antibody response to vaccination, incorporating mean antibody titers, vaccine-specific Ig subclasses, and seroprotection and seroconversion, and sample size calculations are presented in detail in Ref. (26). A total of 62 young subjects and 63 older subjects entered the study and 58 young and 54 older subjects completed the study (26). Two subjects experienced adverse effects (gastrointestinal bloating) during the study, one on the placebo group and one in the *Bifidobacterium longum* + gluco-oligosaccharide (Gl-OS) group; both withdrew from the study.

### Study Design

Subjects were randomized by covariate adaptive randomization (by a research nurse not involved in the study) according to gender, age, and BMI to receive *B. longum bv. infantis* CCUG 52486 (*B. longum*, 10^9^ CFU in 1 g skimmed milk powder/day) combined with [Gl-OS (BioEcolians, Solabia); 8 g/day] in a double-blind, placebo controlled parallel study designed for 8 weeks. After 4 weeks, subjects were administered with a single dose of the influenza vaccine (Influvac^®^subunit 2010/2011 season, Abbott Biologicals B.V., lot number 1070166) containing A/California/7/2009 (H1N1), A/Perth/16/2009 (H3N2) and the B/Brisbane/60/2008-like strain by intramuscular injection in the deltoid, as described in Ref. (26).

### Blood Sample Processing

For serum, blood was collected into serum separator tubes and left at room temperature for 30 min to allow coagulation. Samples were centrifuged at 1,300 × *g* for 10 min and aliquots of serum were collected and stored at −80°C prior to analysis.

### NK Cell Phenotyping

Cryopreserved peripheral blood mononuclear cells (PBMCs) were thawed, washed, counted in a Z1 Coulter Counter, and adjusted to 5 × 10^6^ cells/ml. Cryopreservation has been shown to have no effect on NK cell function (29). Viability was assessed by trypan blue dye exclusion (Sigma, UK) and was typically >85%. Cells were then resuspended in the appropriate medium for phenotyping or functional assays. NK cell phenotyping was performed using the following fluorescent-conjugated monoclonal antibodies: CD3-PE-Cy7, CD56-PE, CD16-FITC, and CD57-APC (BD Biosciences, UK). For determination of non-specific staining, cells were incubated with mouse IgG1 as an isotype negative control for PE-labeled antibodies (BD Biosciences, UK). PBMCs (1 × 10^6^) were incubated with the antibody combination for 20 min in the dark at room temperature before washing and fixing with 2% paraformaldehyde buffer and analysis on a flow cytometer (BD FACS Canto II, BD Bioscience), which was performed within 5 h.

The lymphocyte population was gated using forward scatter and side scatter and NK cells were identified as CD3^−^CD56^+^ (Figure S1 in Supplementary Material). Based on the CD3^−^CD16^+^CD56^+^ phenotype, NK cells were further divided into CD56^bright^ and CD56^dim^ subsets and the proportions of these cells were determined (Figure S2 in Supplementary Material). Expression of CD57^+^ by both the total NK cell population and specific NK cell subsets was also assessed.

Data was analyzed using FlowJo software©Tree star according to the gating strategy described in Figure S3 in Supplementary Material.

### NK Cell Activity

K562 myeloid leukemia cells (target cells for the NK cell activity assay) were enumerated by microscopy with trypan blue exclusion, adjusted to 5 × 10^6^ cells/ml and washed twice with phosphate-buffered saline (PBS) prior to incubation with carboxyfluorescein diacetate *N*-succinimidyl ester (CFDA-SE) for 45 min at 37°C in an air/CO2 (19:1) atmosphere. Following incubation, the K562 cells were washed twice with PBS and resuspended in 1 ml of complete medium comprising RPMI 1640 with l-glutamine, 5% streptomycin, and 10% newborn calf serum.

For the assay, PBMCs were incubated with the CFDA-SE labeled K562 cells for 2 h at 37°C in an air/CO2 (19:1) atmosphere at effector to target cells ratios of 100:1, 50:1, 25:1, and 12.5:1. Samples were then transferred to 4°C and analyzed within 1 h. Propidium iodide (PI) (20 µl) was added to samples immediately prior to analysis on a flow cytometer (FACS Canto II, BD Bioscience with DIVAS software). Data for 1,200 viable target cells was collected and FSC/SSC gating was used to discriminate between leukocytes and K562 target cells. Single parameter histograms representing the FITC channel demonstrate staining of K562 cells by CFDA-SE and of K562 cells within samples with an E/T ratio of 100:1 (Figure S4 in Supplementary Material). Events recorded within the K562 cells gate were further analyzed according to fluorescence of the red DNA dye, PI (PE channel), and fluorescence of CFDA-SE (FITC channel) by gating with quadrant markers, which divide the plot into four sections and enables enumeration of dead and live target cells. NK cell activity was calculated as the difference between the total percentage of lysed target cells and the percentage of lysed K562 cells in the control sample (no PBMC).

### Analysis of Anti-CMV IgG Antibodies

Concentrations of anti-CMV IgG antibodies were analyzed by ELISA according to the manufacturer’s instructions (ab108724 Anti-Cytomegalovirus (CMV) IgG Human Elisa Kit, Abcam, UK) and read in a microplate reader (GENios) at 450 nm, with 620 nm as a reference wavelength. CMV seropositivity, which is often associated with immunosenescence and poor response to vaccination (26), was defined as antibody levels >11 AU/ml in accordance with the manufacturer’s instructions.

### Statistical Analysis

Data were analyzed using SPSS software (version 21). The primary outcome for the trial was the antibody response to vaccination, which has been published (26). For the secondary endpoints of NK phenotype and activity presented in this paper, a linear mixed model (LMM) was implemented. A first order autoregressive covariance structure was selected AR (1), with fixed factors of time (repeated measures), age, and treatment and subject as a random effect. Following this initial analysis, the data were split by cohort (young/older) and the analysis was repeated in the same manner to determine time and treatment effects within each cohort. The distribution of the data was checked using the Kolmogorov–Smirnov test. If data were not normally distributed, they were log transformed. Differences in baseline phenotype and NK activity between the young and older subjects were analyzed by independent samples *t*-tests. Relationships between NK activity and seroconversion were analyzed by the Fisher’s exact test. To account for multiple testing, two-sided *P* values of 0.01 or less were considered statistically significant. All missing data were classed as missing at random and only available data were analyzed.

## Results

### Subject Characteristics

The characteristics of the subjects recruited to the study have been previously reported (26). Of the 125 volunteers who started the trial, 112 completed (26). NK activity analysis was performed on samples from 51 young subjects and 52 older subjects. There were no differences in baseline characteristics, such as age, BMI, blood pressure, etc., between treatment groups within the young or older cohorts.

### Effect of Aging on the NK Cell Repertoire

Baseline NK cell phenotypes were significantly different between the age groups (Table 1). Young subjects had significantly higher numbers of total lymphocytes than older subjects (*P* < 0.001). However, older subjects had significantly higher percentages of CD3^−^CD56^+^ and CD56^−^CD16^+^ cells than young subjects (*P* < 0.01 at least), while percentages of CD3^+^CD56^+^ cells did not differ significantly between young and older subjects.

**Table 1:** Age-related differences in the natural killer (NK) cell population at baseline.

% of Immune cells	Parent population	Young subjects	Older subjects	Age *P*-value
Lymphocytes	Peripheral blood mononuclear cells	79.32 ± 0.87	71.76 ± 1.12	<0.001
CD56^−^CD16^+^	Lymphocyte	5.47 ± 0.52	8.62 ± 0.74	<0.01
CD3^+^CD56^+^	Lymphocyte	3.70 ± 0.63	4.05 ± 0.87	NS
CD3^+^CD56^−^	Lymphocyte	67.3 ± 1.84	69.1 ± 1.24	NS
CD3^−^CD56^+^	CD3^−^ lymphocyte	16.9 ± 1.26	24.3 ± 1.25	<0.001
CD56^bright^	CD3^−^CD56^+^	9.88 ± 1.02	6.86 ± 0.69	0.016
CD56^bright^CD16^−^	CD3^−^CD56^+^	8.69 ± 0.92	5.68 ± 0.57	<0.01
CD56^bright^CD16^dim^	CD3^−^CD56^+^	1.81 ± 0.22	± 0.19	NS
CD56^dim^	CD3^−^CD56^+^	89.37 ± 1.06	92.61 ± 0.72	0.013
CD56^dim^CD16^−^	CD3^−^CD56^+^	28.79 ± 1.51	20.17 ± 1.36	<0.001
CD56^dim^CD16^+^	CD3^−^CD56^+^	60.72 ± 2.04	72.51 ± 1.73	<0.001
CD3^+^CD57^+^	CD3^+^	8.16 ± 0.62	11.57 ± 0.91	<0.01
CD3^−^CD56^+^CD57^+^	CD3^−^CD56^+^	46.12 ± 1.91	50.56 ± 1.82	NS
CD3^+^CD56^+^CD57^+^	CD3^+^CD56^+^	41.65 ± 2.74	54.08 ± 2.80	<0.01
CD56^dim^CD57^−^	CD56^dim^	40.80 ± 1.33	35.93 ± 1.49	0.017
CD56^dim^CD57^+^	CD56^dim^	45.50 ± 1.80	52.57 ± 1.82	<0.01
CD56^dim^CD16^+^CD57^+^	CD56^dim^CD16^+^	56.70 ± 1.84	60.44 ± 1.87	NS
CD56^dim^CD16^−^CD57^+^	CD56^dim^CD16^−^	18.02 ± 1.29	19.69 ± 1.58	NS

*Data are mean ± SEM for *n* = 51 young subjects and *n* = 52 older subjects. Differences between the two cohorts were analyzed by independent samples *t*-tests (2-tailed)*.

CD56^bright^ and CD56^dim^ cells were expressed as percentages of the CD3^−^CD56^+^ NK cell pool. Older subjects tended to have a higher proportion of CD56^dim^ NK cells (*P* = 0.013) and a lower proportion of CD56^bright^ cells (*P* = 0.016) than young subjects. Further analysis of the CD56^dim^ population demonstrated that there was a significantly higher percentage of CD56^dim^CD16^+^ NK cells (*P* < 0.001), and a lower percentage of CD56^dim^CD16^−^ NK cells (*P* < 0.001) in older subjects compared to young subjects. Further analysis of the CD56^bright^ population demonstrated that older subjects had a lower proportion of CD56^bright^CD16^−^ cells than young subjects (*P* < 0.01). Overall, therefore, the higher proportion of CD56^dim^ cells in older subjects was mainly reflected in the CD16^+^ subset, whereas the lower proportion of CD56^bright^ cells was mainly reflected in the CD16^−^ subset. There was no difference in the percentage of CD56^bright^CD16^dim^ cells between the cohorts (Table 1).

### NK Cell Populations and Markers of Immunosenescence

Older subjects expressed higher levels of the aging marker, CD57 on the CD3^+^, CD3^+^CD56^+^, and the CD56^dim^ populations (*P* < 0.01 for both) compared to young subjects (Table 1). In contrast, CD57 expression on the CD3^−^CD56^+^, CD56^dim^CD16^+^, and CD56^dim^CD16^−^ populations was not significantly different between the two cohorts.

**Figure 1:**
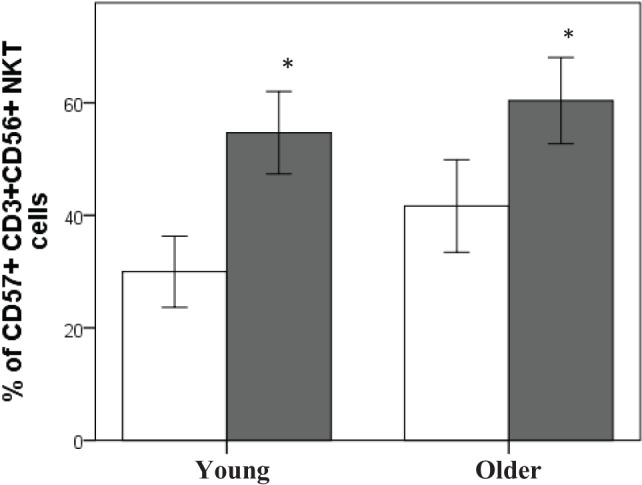
CMV seropositivity is associated with an increase in CD57^+^CD3^+^CD56^+^NKT cells. Data are % of CD57^+^CD3^+^CD56^+^ NKT cells ± SEM for *n* = 40 young and *n* = 41 older subjects, ▫CMV^−^, ▪CMV^+^. Data were analyzed using independent samples *t*-test. *Denotes significant difference between CMV and CMV^+^ status in young (*P* < 0.001) and older subjects (*P* < 0.01).

Subjects who were seropositive for CMV had a significantly higher percentage of CD57^+^CD3^+^CD56^+^ NKT cells compared to subjects who were CMV^−^, regardless of age (*P* < 0.01 at least) (Figure 1).

### Effect of Aging on NK Cell Activity

**Figure 2:**
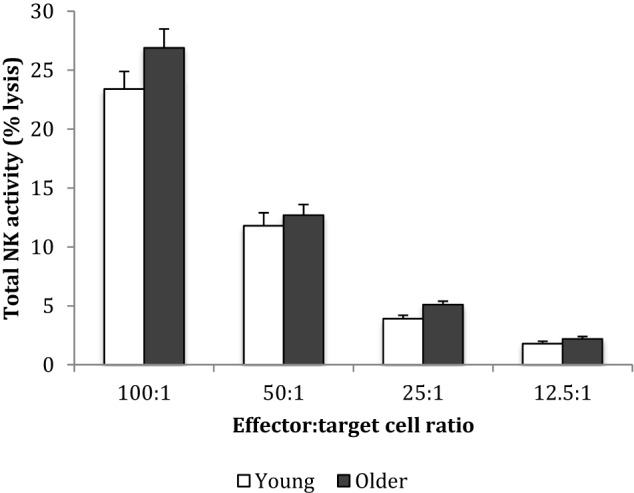
Baseline natural killer cell activity. Data are mean ± SEM at week 0, for *n* = 54 subjects per group. Data were analyzed using independent samples *t*-test (2-tailed). *P* = 0.11 for E/T ratio 100:1, *P* = 0.54 for 50:1, *P* = 0.03 for 25:1, and *P* = 0.08 for 12.5:1.

**Figure 3:**
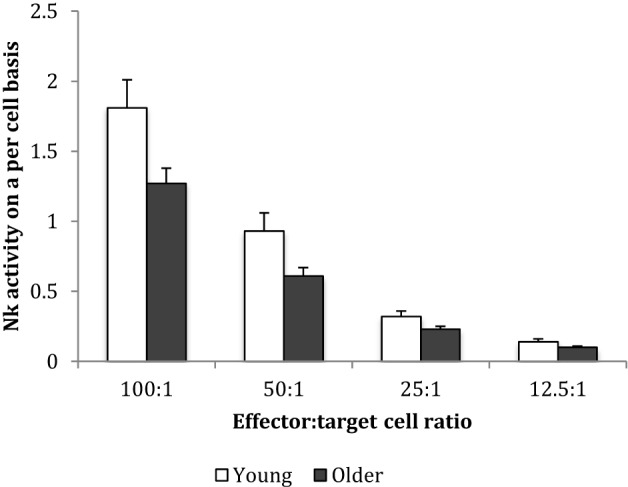
Baseline natural killer cell activity on a per cell basis. Data are mean ± SEM at week 0, for *n* = 54 subjects per group. Data were analyzed using independent samples *t*-test (2-tailed). *P* = 0.02 for E/T ratio 100:1, *P* = 0.03 for 50:1, *P* = 0.038 for 25:1, and *P* = 0.116 for 12.5:1.

Baseline NK cell activity was not different between young and older subjects at any of the E/T ratios (Figure 2). However, there were trends for higher baseline NK cell activity on a per cell basis (% NK activity expressed relative to % CD3^−^CD56^+^ NK cells) in the young subjects compared to the older subjects at E/T ratios of 100:1, 50:1, 25:1 (Figure 3).

### Effect of *B. longum* + Gl-OS on NK Cell Phenotype and Activity

Supplementation with *B. longum* + Gl-OS had no significant effect on NK cell phenotype (Tables S1A,B in Supplementary Material) or activity (Table 2; Tables S2A,B in Supplementary Material) in young or older subjects, either before or after vaccination.

**Table 2:** Natural killer (NK) cell activity before and after the treatment with *Bifidobacterium longum* + gluco-oligosaccharide (Gl-OS).

Young cohort							Older cohort					
	Baseline	Week 4	Week 6	Week 8	*P-*value week 0 vs 4	*P-*value LMM	Baseline	Week 4	Week 6	Week 8	*P-*value week 0 vs 4	*P-*value LMM
**Total NK cell activity**												
Synbiotic	25.05 ± 2.19	25.32 ± 1.80	27.23 ± 1.85	29.82 ± 2.28	0.894	0.249	27.64 ± 2.55	31.79 ± 2.77	31.28 ± 2.86	32.97 ± 2.09	0.015	0.093
Placebo	21.43 ± 1.58	22.69 ± 2.24	25.93 ± 2.37	25.72 ± 2.03	0.145	0.249	26.42 ± 2.18	28.34 ± 2.25	26.54 ± 1.95	27.44 ± 1.80	0.316	0.093

**NK cell activity on a per cell basis**												
Synbiotic	1.97 ± 0.28	2.29 ± 0.36	2.22 ± 0.33	2.54 ± 0.42	0.183	0.657	1.22 ± 0.16	1.33 ± 0.18	1.41 ± 0.17	1.42 ± 0.16	0.203	0.868
Placebo	1.62 ± 0.24	1.84 ± 0.29	2.18 ± 0.40	2.43 ± 0.39	0.173	0.657	1.30 ± 0.15	1.38 ± 0.17	1.38 ± 0.15	1.28 ± 0.15	0.209	0.868

*Data are mean (±2 SEM) for *n* = 32 and 25 subjects per group at E/T ratio 100:1 (for full data at all E/T ratios, please see Tables S2A,B in Supplementary Material). Data were analyzed using paired samples *t*-tests (before vaccination) and linear mixed model (LMM) (after vaccination). There were no statistically significant effects of *B. longum* + Gl-OS at any E/T ratio*.

### Effect of Influenza Vaccination on NK Cell Phenotype and Activity

Since there was no effect of the synbiotic on NK cell phenotype, either before or after vaccination, treatment groups are presented combined to allow evaluation of the effects of aging and vaccination. Vaccination significantly decreased the proportion of CD3^−^CD56^+^ and CD3^−^CD56^+^CD57^+^ NK cells (relative to CD3^−^ lymphocytes) following vaccination in older subjects, but not young subjects (LMM, older cohort only, effect of time *P* < 0.01) (Table 3). In contrast, in the young subjects, vaccination resulted in an increase in CD56^bright^ cells and a decrease in CD56^dim^ cells (LMM, young cohort only, effect of time *P* < 0.01) (Table 3). Within the CD56^bright^ population, there was an increase in the percentage of CD56^bright^CD16^dim^ NK cells in young subjects following vaccination (LMM, effect of time *P* < 0.01) (Table 3).

**Table 3:** Changes in natural killer cell phenotype following influenza vaccination in young and older subjects.

		Effect of vaccination		

% of Immune cells	Parent population	Both cohorts (*P*-value)	Young cohort (*P*-value)	Older cohort (*P*-value)
Lymphocytes	Peripheral blood mononuclear cells	NS	NS	NS
CD3^−^CD56^+^	Lymphocyte	↓*P* = 0.017	NS	↓*P* < 0.01
CD56^−^CD16^+^	Lymphocyte	NS	NS	NS
CD3^+^CD56^+^	Lymphocyte	NS	NS	NS
CD3^+^	Lymphocyte	NS	NS	NS
CD3^+^CD57^+^	CD3^+^	NS	NS	NS
CD56^bright^	CD3^−^CD56^+^	↑*P* < 0.01	↑*P* < 0.01	NS
CD56^bright^CD16^−^	CD3^−^CD56^+^	NS	NS	NS
CD56^bright^CD16^dim^	CD3^−^CD56^+^	↑*P* = 0.015	↑*P* < 0.01	NS
CD56^dim^	CD3^−^CD56^+^	↓*P* < 0.01	↓*P* < 0.01	NS
CD56^dim^CD16^−^	CD3^−^CD56^+^	NS	NS	NS
CD56^dim^CD16^+^	CD3^−^CD56^+^	NS	NS	NS
CD3^−^CD56^+^CD57^+^	CD3^−^CD56^+^	↓*P* = 0.029	NS	↓*P* < 0.01
CD3^+^CD56^+^CD57^+^	CD3^+^CD56^+^	NS	NS	NS
CD56^dim^CD57^−^	CD56^dim^	NS	NS	NS
CD56^dim^CD57^+^	CD56^dim^	NS	NS	NS
CD56^dim^CD16^+^CD57^+^	CD56^dim^CD16^+^	NS	NS	NS
CD56^dim^CD16^−^CD57^+^	CD56^dim^CD16^−^	NS	NS	NS

*Data were analyzed using a linear mixed model with fixed factors of time, age, and treatment. In further analysis, data were split by cohorts, which were analyzed separately*.

### Post-Vaccination NK Cell Activity Is Associated With Greater Seroconversion to the Brisbane Subunit of the Influenza Vaccine

Subjects were classified as exhibiting “low” or “high” NK cell activity by using the median as a cut-off (24% lysis for young subjects and 29% for older subjects). For older subjects, there were no seroconverters to the Brisbane strain and to all three strains of the influenza vaccine in the low NK activity group, whereas in the high NK activity group, 27% of subjects seroconverted to the Brisbane strain (Fisher’s exact test, *P* = 0.01) and all strains of the vaccine (Fisher’s exact test, *P* = 0.01), suggesting that high post-vaccination total NK activity was associated with a greater rate of seroconversion. This relationship was not observed in the young subjects. There were no associations between NK activity and antibody response to H1N1 or H3N2 subunits of the vaccine in either of the cohorts.

## Discussion

This study highlights phenotypic changes in the NK cell population during aging, the most prominent of which were an increase in CD56^dim^ cells, mainly reflected in the CD16^+^ subset, a decrease in CD56^bright^ cells, mainly reflected in the CD16^−^ subset, and greater expression of the immunosenescence marker, CD57, on some NK cell subsets. It also demonstrates that CMV positivity, often suggestive of repeated antigenic exposure, was associated with an increase in an immunosenescent population of NKT cells. We previously reported that higher plasma levels of anti-CMV IgG in the older subjects were associated with higher numbers of senescent (CD28^−^CD57^+^) helper T cells and a failure of these subjects to seroconvert to the Brisbane subunit of the vaccine (26). We also reported that antigen-specific B and T cell activation following an *in vitro* recall challenge with the influenza vaccine was influenced by CMV seropositivity (30). The changes in NK cell phenotype only partially translated to differences in NK cell activity, observed as trends toward reduced NK cell activity in older subjects when analyzed on a per cell basis. The effects of influenza vaccination on NK cell phenotype and activity were modest and less evident in the older subjects than the young subjects. Furthermore, higher post-vaccination NK activity was associated with better seroconversion in the older subjects. There was no effect of the synbiotic on NK cell phenotype or activity, either before or after influenza vaccination.

While there is general consensus that aging impairs the response to influenza vaccination (31), there are very few robust studies specifically comparing responses of young and older subjects, and the most comprehensive information available is from a meta-analysis published in 2006, which concluded that clinical vaccine efficacy in older subjects was only 17–53% compared to 70–90% in young subjects (32). In this study, antibody responses to all three subunits of the influenza vaccine were impaired in the older subjects and there was significantly reduced seroprotection to the H1N1 and Brisbane subunits and seroconversion to the H3N2 and Brisbane subunits after vaccination; these data are reported elsewhere (26). The expansion of the mature CD56^dim^ NK cell population and decline of the immature CD56^bright^ NK cell population could contribute to diminished adaptive immune responses to vaccination in older subjects, since CD56^bright^ NK cells drive DC maturation and T cell differentiation (33). Expansion of the CD56^−^CD16^+^ NK cell population in older subjects, also observed in this study, has been associated with low cytotoxic capacity, low cytokine production, and exposure to chronic viral infections (34). Expression of CD57, a marker for terminally differentiated cells, was increased with the CD3^−^CD56^+^ NK cell population of older subjects, and this effect of aging is consistent with some (17, 20, 34), but not all, previous reports (18). Thus, there are a number of potentially important aspects to remodeling of the NK cell population during the life course, which could diminish the response to vaccination. However, there is less clarity about the impact of aging on NK cell activity.

The alterations in NK cell phenotype due to aging only partially resulted in impairment of NK cell activity in the older subjects; this was evidenced by a trend toward decreased NK activity on a per cell basis in the older subjects, but total NK activity was not reduced. Overall, therefore, NK cell activity appears to be relatively well preserved during aging, despite potentially unfavorable changes in NK phenotype. CD56^+^CD57^+^ cells, which characteristically have high cytolytic capacity, but diminished proliferation, were evident in the older subjects and may contribute to the preservation of NK cell activity in old age. NK cell activity normally remains elevated for up to 30 days following influenza vaccination (9). One study in older subjects reported no effect of vaccination on NK cell activity for 4–6 weeks of post-vaccination (10), but it is not clear whether this was due to a return to baseline NK activity at these later time points or because NK cells failed to respond to vaccination in older subjects. Thus, a direct comparison of NK cell activity in young and older subjects in response to influenza vaccination is timely and important.

This study is the first to demonstrate that older subjects with high NK activity post-vaccination tended to seroconvert better to the Brisbane strain and all influenza strains together compared to subjects with low NK activity; these effects were borderline significant. While this cannot be taken to imply causality, it builds on evidence suggesting an association between high NK activity and antibody response to influenza vaccination in both young (35) and elderly subjects (9). To our knowledge, this study is the first to directly and comprehensively compare NK cell phenotype and activity in response to influenza vaccination in young and older subjects. To date, only three human studies have investigated NK cell numbers in response to influenza vaccine and reported either no change in elderly (10) or young individuals (4), or an initial decrease in numbers of CD3^−^CD56^+^ cells in adults, with a subsequent increase 4 days after seasonal and pandemic H1N1 influenza vaccine (36). However, these studies were small, did not directly compare responses in young and older subjects and employed different blood sampling times. In this study, the decrease in NK cells in older subjects 14 days after vaccination was profound, but there was some recovery at 28 days (data not shown). This is consistent with a report demonstrating an initial decline in the number of NK cells during 2 weeks following H1N1 influenza vaccination, with later recovery (37). This study is also the first to demonstrate that influenza vaccination was associated with an increase in the percentage of CD56^bright^ cells in young subjects, including CD56^bright^CD16^−^ and CD56^bright^CD16^dim^ subsets, whereas the percentage of CD56^dim^ cells was reduced 2 weeks after vaccination. This increased proportion of CD56^bright^ cells in young adults could be related to peripheral expansion, increased production from NK precursors in the bone marrow, or redistribution from the tissues after vaccination (16), and could contribute to DC maturation and stimulation of T cells. Further work in larger studies to allow disaggregation by gender and race could be of value; there was insufficient power to produce meaningful data in this study.

Enhanced NK cell activity as a result of treatment with probiotics is commonly reported, both *in vitro* (38, 39), and in human studies (40, 41) and it is suggested that this effect might be strain-specific. This study tested the hypothesis that the probiotic *B. longum bv. infantis* CCUG 52486 combined with prebiotic, Gl-OS, would improve NK cell activity in an age-dependent manner, as a secondary outcome of the PRIMAGE study [the primary outcome being antibody production (26)]. The strain *B. longum bv. infantis* CCUG 52486 was selected because it was originally isolated from a cohort of very healthy elderly subjects (independent lifestyle, free of chronic disease, and aged 90 years or over) in Italy as part of the CROWNALIFE EU FP5 project (42), it has been demonstrated to have particular ecological fitness and anti-pathogenic effects *in vitro* (43), and it has immunomodulatory effects which are strongly influenced by the age of the host (39). However, there were no effects of the synbiotic on NK cell phenotype or activity, either prior to or after vaccination. Close scrutiny of the literature reveals that while some studies demonstrate enhancement in both the proportion of NK cells (CD16^+^CD56^+^) and NK activity by probiotics (44), some demonstrate only enhancement in the proportion of NK cells (45, 46), and others demonstrate no effect at all (47–49). Supplementation with *L. paracasei* NCC 2461, prebiotic fructo-oligosaccharides, and a range of vitamins and nutrients for 4 months enhanced pre-vaccination NK cell activity and NK cell numbers and reduced the number of infections in elderly Chilean subjects (50). In contrast, De Vrese et al. (51) reported reduced duration of cold episodes, but no effect on NK cell numbers in healthy adults following intervention with a mixed supplement, including *L. gasseri* PA 16/8, *B. longum* SP 07/3, *B. bifidum* MF 20/5, vitamins, and minerals for 3 months (52). Thus, it is not clear whether the impact of pre- and probiotics on infection and illness relates to changes in NK cell number or activity. The effects of probiotics on response to influenza vaccination in older adults are of particular importance, given the consequences of respiratory infections in older people (53); however, it is possible that not only there are differences in the immunomodulatory potential of different strains, but also there is differential immune response to probiotics in young vs older subjects. You et al. (39) demonstrated that PBMC from older subjects (60–85 years) were more responsive to the immunoregulatory effects (IL-10 induction) of two strains of bifidobacteria than young subjects (18–30 years), whereas PBMC from young subjects were more responsive to the immunostimulatory effects (IL-12 induction) of two strains of lactobacilli. Further studies demonstrated that probiotics (including the strain employed in this study) increased the responsiveness of DCs in older subjects to a greater degree than young subjects, but this was not sufficient to overcome the impact of immunosenescence in a mixed leukocyte reaction (54).

The results of an intervention should, if possible, take into account the potential influence of baseline differences, for example in immunosenescence and pre-vaccination antibody titers. Here, there are some limitations; we previously reported that the older subjects randomized to the synbiotic had, by chance, a significantly higher number of senescent (CD28^−^CD57^+^) helper T cells and higher plasma levels of anti-CMV IgG at baseline compared to those randomized to the placebo, placing them at a significant disadvantage before vaccination and potentially influencing the outcome of the trial (26). Attempts to take into account pre-existing immunity to influenza is challenging. There was virtually no seroprotection to the H1N1, H3N2, or Brisbane subunits exhibited by the subjects prior to vaccination, with the exception of a small number of young subjects who appeared to have been exposed to the H1N1 subunit, but did not report infection (26). There was also no significant difference in pre-vaccination antibody titers to the H1N1, H3N2, or Brisbane strains between young and older subjects (26). We did not assess subjects for pre-existing immunity to other influenza strains, including those that had been employed in influenza vaccination in previous years for logistical reasons. However, prior exposure may well have influenced the outcome of vaccination, since cross-reactivity between influenza viruses is well documented (55) and pre-existing immunity to flavivirus has been demonstrated to influence the response to yellow fever vaccination (56). Future work characterizing in detail the influence of both pre-existing immunosenescence and pre-existing immunity on vaccination outcome is warranted.

In conclusion, this study describes marked alteration of the NK cell population by aging, although NK cell activity was preserved to some degree in older subjects. Although there was evidence of an impaired NK cell response to influenza vaccination in older subjects, this was not offset by *B. longum* + Gl-OS. Further work to examine the influence of pre-existing immunity on outcomes of vaccination trials would be of value.

## Ethics Statement

The study protocol was reviewed and approved by the University of Reading Research Ethics Committee (project number: 10/09) the National Health Service (NHS) Research Ethics Committee for Wales (10/MRE09/5). The trial was registered with clinicaltrials.gov (Identifier: NCT01066377) and conducted according to the guidelines laid down in the Declaration of Helsinki.

## Author Contributions

PY, KT, ST, and MG designed the research. AP-K, CC, CM, and HD conducted the research. AP-K, CC, and PY analyzed the data. PY and AP-K wrote the first draft of the paper. All authors contributed to the final manuscript, approved the final version, and agreed to be accountable for the content of the work.

## Conflict of Interest Statement

The authors declare that the research was conducted in the absence of any commercial or financial relationships that could be construed as a potential conflict of interest.

## Abbreviations

BSA, bovine serum albumin; CIRS, cumulative illness rating scale; Gl-OS, gluco-oligosaccharide; Ig, immunoglobulin; PBMC, peripheral blood mononuclear cells; NK, natural killer; PBS, phosphate-buffered saline; RPMI, Roswell Park Memorial Institute.
