# Supplementary material for: Age-Related Changes in the Natural Killer Cell Response to Seasonal Influenza Vaccination Are Not Influenced by a Synbiotic: a Randomised Controlled Trial
Source: Front Immunol. 2018 Mar 22;9:591. doi: 10.3389/fimmu.2018.00591 (PMC5890114; doi:10.3389/fimmu.2018.00591)
Supplement: Supplementary file 1 [file presentation_1.PDF]

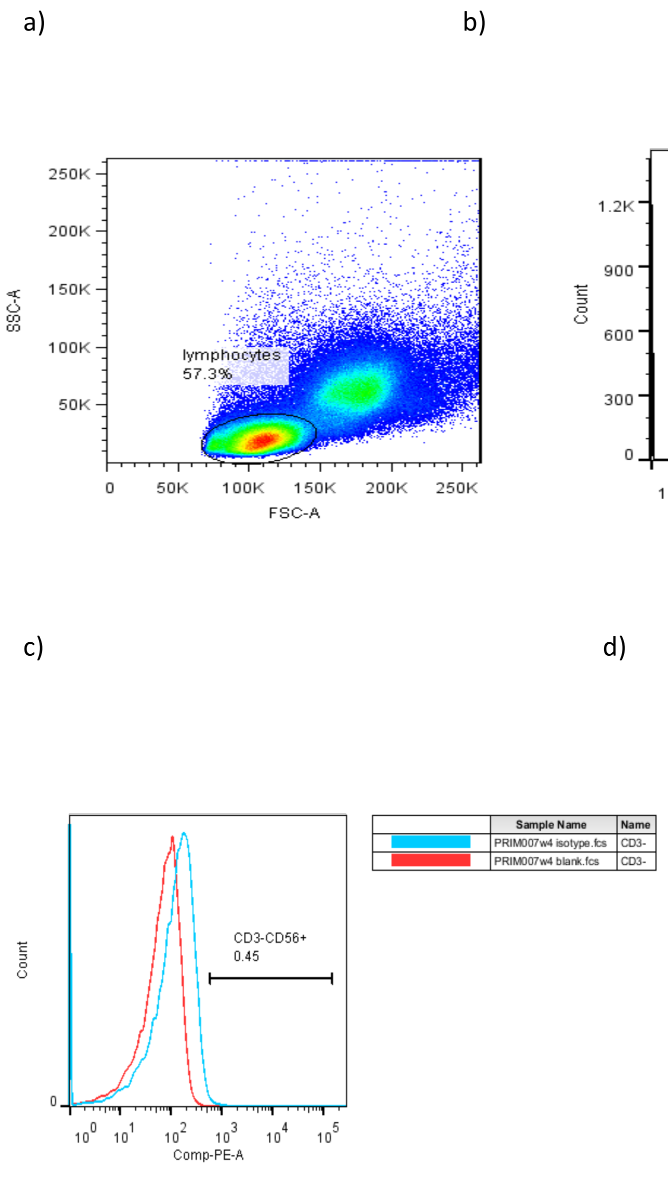

Supplementary Figure 1. Gating of a) lymphocytes; b) CD3$^-$ cells; c) isotype control for CD3$^-$CD56$^+$; d) CD3$^-$CD56$^+$ cells.

a) Dot plot of leukocytes in PBMC sample (FSC-A vs SSC-A). Lymphocytes are displayed on the left and monocytes on the right. b) The horizontal axis displays the CD3 T cell signal and the vertical axis shows the number of events. The negative peak corresponds to CD3$^-$ cells. c) The horizontal axis displays the isotype control and blank signal. The gate is placed to capture CD56$^+$ cells. d) CD3$^-$CD56$^+$ cells (PE- positive); the horizontal axis displays the CD3$^-$CD56$^+$ signal and the vertical axis shows the number of events. The positive peak corresponds to CD56$^+$ cells.

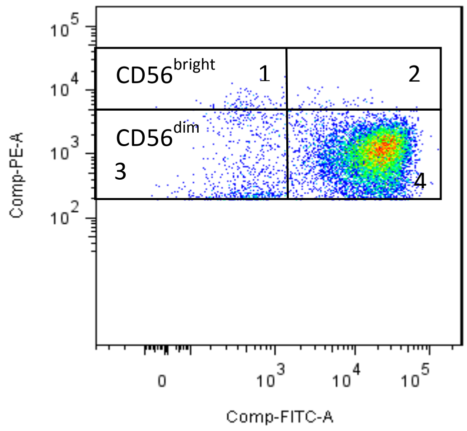

Supplementary Figure 2. Identification of % CD56^{bright} and CD56^{dim} populations. The upper-left quadrant corresponds to CD56^{bright}CD16^{-} cells, the upper-right quadrant corresponds to CD56^{bright}CD16^{dim} cells, the lower-left quadrant corresponds to CD56^{dim}CD16^{-} cells and the lower-right quadrant corresponds to CD56^{dim}CD16^{+} cells.

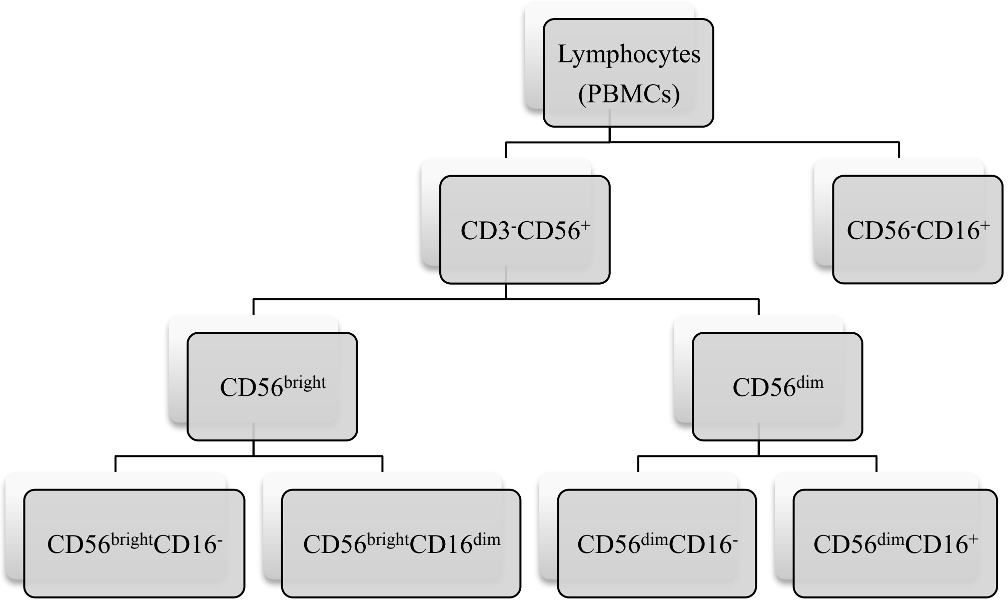

Supplementary Figure 3. Gating strategy for NK cell phenotyping.

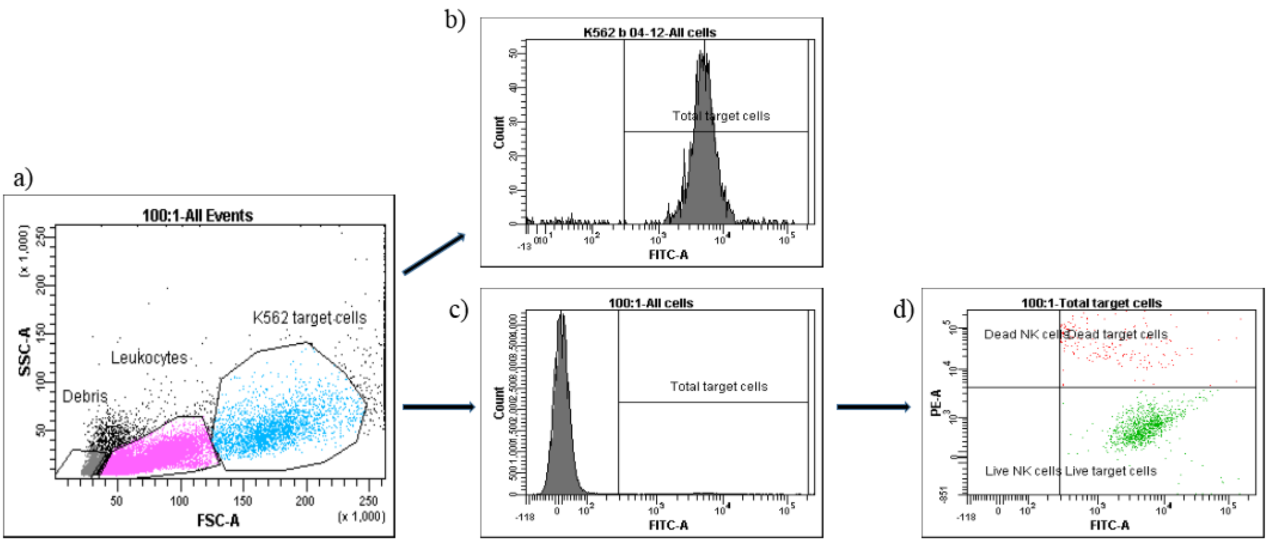

Supplementary Figure 4. Gating strategy for NK cell activity assay a) FSC/SSC dot plot of PBMCs and viable K562 target cells. Staining of b) K562 target cells c) PBMCs and K562 target cells at a 100:1 ratio d) Flow cytometric analysis of dead target cells, which represent NK cell activity. The upper-right quadrant contains events positive for red and green fluorescence PE^+ $FITC^+$ (dead target cells), whereas the lower-right quadrant shows live target cells.
