# Supplementary material for: Age-Related Changes in the Natural Killer Cell Response to Seasonal Influenza Vaccination Are Not Influenced by a Synbiotic: a Randomised Controlled Trial
Source: Front Immunol. 2018 Mar 22;9:591. doi: 10.3389/fimmu.2018.00591 (PMC5890114; doi:10.3389/fimmu.2018.00591)
Supplement: Supplementary file 2 [file presentation_2.PDF]

Supplementary Table 1A. Effect of placebo on proportion of NK cell subsets in young and older cohort. Data are mean ± SEM for n= 25 young subjects and n= 27 older subjects on placebo treatment. Data were analysed using a paired samples t –test (before vaccination) and using a Linear Mixed Model (after vaccination).

**% of immune cells**	**Parent population**	**Young cohort**						**Older cohort**					
		**baseline**	**week 4**	*P*- value Wk0vs4	**Week 6**	**Week 8**	*P*- value LMM	**baseline**	**week 4**	*P*- value Wk0vs4	**Week 6**	**Week 8**	*P*- value LMM
Lymphocytes	PBMC	78.00±1.18	78.59±1.00	0.456	78.45±1.25	79.01±1.28	0.840	71.56±1.85	71.71±1.56	0.912	70.97±2.09	73.21±1.51	0.790
$CD56^{-}$ $CD16^{+}$	lymphocyte	5.99±0.94	5.57±0.71	0.496	5.27±0.74	4.91±0.58	0.120	8.511±0.92	7.99±0.68	0.254	8.60±0.98	8.79±0.83	0.114
$CD3^{+}CD56^{+}$	lymphocyte	3.15±0.43	3.02±0.44	0.289	2.81±0.39	2.80±0.43	0.271	3.02±0.87	3.17±0.90	0.115	3.28±0.96	3.20±0.89	0.192
$CD3^{+}$ $CD56^{-}$	lymphocyte	67.67±1.91	67.49±1.90	0.892	68.35±1.35	71.43±1.20	0.468	69.87±1.44	69.39±1.28	0.713	68.92±1.35	70.28±1.30	0.782
$CD3^{-}CD56^{+}$	$CD3^{-}$ lymphocyte	16.91±1.26	16.40±1.65	0.121	16.33±1.72	14.87±1.35	0.141	24.28±1.25	24.74±1.84	0.835	23.12±1.75	25.23±1.83	0.335
$CD56^{bright}$	$CD3^{-}CD56^{+}$	11.10±1.74	10.40±1.74	0.259	11.63±2.03	10.97±1.69	0.975	6.79±1.00	6.87±0.81	0.902	7.61±0.94	6.91±0.99	0.156
$CD56^{bright}CD16^{-}$	$CD3^{-}CD56^{+}$	9.84±1.62	9.33±1.60	0.327	10.03±1.72	9.92±1.61	0.443	5.68±0.87	5.82±0.71	0.819	6.35±0.84	5.79±0.89	0.571
$CD56^{bright}CD16^{dim}$	$CD3^{-}CD56^{+}$	1.88±0.32	1.55±0.30	0.040	2.24±0.42	1.71±0.26	0.407	1.58±0.27	1.54±0.22	0.833	1.76±0.23	1.60±0.25	0.479
$CD56^{dim}$	$CD3^{-}CD56^{+}$	88.16±1.80	88.97±1.78	0.205	87.57±2.10	88.15±1.76	0.936	92.67±1.04	92.59±0.84	0.911	91.82±0.98	92.56±1.04	0.090
$CD56^{dim}CD16^{-}$	$CD3^{-}CD56^{+}$	27.00±2.20	28.16±2.20	0.246	27.10±2.32	29.71±2.54	0.188	21.15±2.14	22.70±2.21	0.320	22.35±2.37	20.51±2.02	0.407
$CD56^{dim}CD16^{+}$	$CD3^{-}CD56^{+}$	61.30±3.16	60.95±3.11	0.759	60.62±3.06	58.67±3.41	0.343	71.58±2.69	69.93±2.55	0.388	69.54±2.87	72.11±2.69	0.583
$CD3^{-}CD56^{+}CD57^{+}$	$CD3^{-}CD56^{+}$	44.76±3.04	44.60±3.29	0.872	42.85±2.90	42.28±2.83	0.742	50.43±2.77	50.57±2.81	0.880	48.52±2.72	50.95±2.66	0.862
$CD3^{+}CD56^{+}CD57^{+}$	$CD3^{+}CD56^{+}$	40.40±3.30	37.97±3.45	0.050	39.53±3.48	35.79±3.36	0.126	50.18±3.62	48.56±3.97	0.362	48.65±4.06	50.88±4.04	0.145
$CD3^{+}$ $CD57^{+}$	$CD3^{+}$	7.65±0.82	7.94±1.09	0.462	7.13±0.65	6.74±0.61	0.278	11.10±1.01	10.88±1.11	0.671	11.66±1.33	11.71±1.14	0.242
$CD56^{dim}CD57^{-}$	$CD56^{dim}$	41.65±2.22	41.89±2.29	0.774	42.00±2.03	43.56±1.99	0.755	36.76±2.24	35.90±2.38	0.281	37.23±2.21	36.45±2.17	0.677
$CD56^{dim}CD57^{+}$	$CD56^{dim}$	44.83±2.89	45.19±3.08	0.694	44.58±2.76	42.39±2.68	0.504	50.74±2.68	52.20±2.95	0.191	50.49±2.72	51.54±2.73	0.409
$CD56^{dim}CD16^{+}CD57^{+}$	$CD56^{dim}CD16^{+}$	55.24±2.89	55.86±3.04	0.450	55.62±2.93	54.20±2.69	0.407	59.02±2.70	60.68±2.99	0.054	58.90±2.88	59.26±2.72	0.334
$CD56^{dim}CD16^{-}CD57^{+}$	$CD56^{dim}CD16^{-}$	18.38±2.15	18.58±2.40	0.860	16.34±1.89	15.01±1.48	0.889	17.97±1.98	20.98±2.39	0.047	18.85±2.20	19.02±2.40	0.847

Supplementary Table 1B. Effect of *B.longum* + GI-OS on proportion of NK cell subsets in young and older cohort. Data are mean ± SEM for n= 26 young subjects and n= 23 older subjects on *B.longum* treatment. Data were analysed using a paired samples t –test (before vaccination) and using a Linear Mixed Model (after vaccination).

**% of immune cells**	**Parent population**	**Young cohort**						**Older cohort**					
		**baseline**	**week 4**	*P*- value Wk0vs4	**Week 6**	**Week 8**	*P*- value LMM	**baseline**	**week 4**	*P*- value Wk0vs4	**Week 6**	**Week 8**	*P*- value LMM
Lymphocytes	PBMC	80.60±1.23	80.37±1.10	0.769	79.78±1.13	79.96±1.15	0.325	71.90±1.36	69.64±1.84	0.054	71.74±1.97	70.08±1.74	0.335
$CD56^{-}$ $CD16^{+}$	lymphocyte	4.98±0.48	4.57±0.37	0.306	4.52±0.32	4.44±0.37	0.337	8.97±1.28	10.25±1.28	0.031	7.66±1.02	9.12±1.29	0.600
$CD3^{+}CD56^{+}$	lymphocyte	4.23±1.18	3.46±0.98	0.213	3.61±1.02	3.54±1.03	0.608	5.42±1.66	5.57±1.64	0.557	5.40±1.8	4.94±1.23	0.226
$CD3^{+}$ $CD56^{-}$	lymphocyte	69.66±1.70	70.26±1.78	0.650	70.84±1.64	70.60±1.71	0.532	67.60±2.18	67.43±2.06	0.872	67.29±2.33	66.99±1.92	0.287
$CD3^{-}CD56^{+}$	$CD3^{-}$ lymphocyte	16.91±1.26	15.17±1.01	0.218	13.94±1.49	13.95±1.28	0.338	20.63±0.95	26.00±1.36	0.018	24.23±1.62	26.00±1.62	0.601
$CD56^{bright}$	$CD3^{-}CD56^{+}$	8.70±1.08	9.96±1.41	0.126	10.16±1.13	9.22±1.31	0.500	7.02±1.03	6.35±0.98	0.250	6.89±1.05	7.84±1.10	0.700
$CD56^{bright}CD16^{-}$	$CD3^{-}CD56^{+}$	7.58±0.89	8.76±1.19	0.117	8.84±0.92	8.13±1.13	0.443	5.72±0.82	5.21±0.75	0.227	5.69±0.90	6.21±0.89	0.860
$CD56^{bright}CD16^{dim}$	$CD3^{-}CD56^{+}$	1.74±0.30	1.86±0.33	0.339	2.00±0.31	1.70±0.29	0.840	1.74±0.29	1.62±0.34	0.516	1.61±0.29	2.12±0.37	0.407
$CD56^{dim}$	$CD3^{-}CD56^{+}$	90.52±1.13	89.23±1.47	0.133	89.01±1.18	90.01±1.37	0.526	92.47±1.09	93.11±1.04	0.280	92.58±1.09	91.58±1.15	0.685
$CD56^{dim}CD16^{-}$	$CD3^{-}CD56^{+}$	30.52±2.06	32.23±2.01	0.078	32.72±1.92	34.82±2.33	0.109	19.25±1.75	19.66±2.29	0.712	19.97±2.14	19.12±1.95	0.470
$CD56^{dim}CD16^{+}$	$CD3^{-}CD56^{+}$	60.17±2.65	57.15±2.71	0.020	56.44±2.49	55.35±3.01	0.418	73.29±2.32	73.51±2.70	0.865	72.74±2.66	72.55±2.52	0.666
$CD3^{-}CD56^{+}CD57^{+}$	$CD3^{-}CD56^{+}$	47.48±2.34	45.26±2.15	0.087	44.44±2.34	44.76±2.40	0.742	50.40±2.57	51.87±2.69	0.162	51.28±2.50	51.05±2.66	0.862
$CD3^{+}CD56^{+}CD57^{+}$	$CD3^{+}CD56^{+}$	42.89±4.42	43.74±4.23	0.572	42.71±4.33	42.62±4.28	0.334	56.26±4.32	56.0±4.26	0.862	58.42±4.44	56.30±4.13	0.278
$CD3^{+}$ $CD57^{+}$	$CD3^{+}$	9.00±0.91	9.15±1.07	0.795	9.45±1.12	9.25±1.02	0.295	12.36±1.68	12.97±1.79	0.508	11.84±1.82	12.61±1.63	0.501
$CD56^{dim}CD57^{-}$	$CD56^{dim}$	39.99±1.54	41.41±1.28	0.183	42.03±1.47	43.39±1.74	0.942	34.7±2.12	34.65±2.18	0.956	35.07±2.11	34.60±2.09	0.620
$CD56^{dim}CD57^{+}$	$CD56^{dim}$	46.15±2.21	44.62±1.78	0.240	43.75±2.11	42.86±2.31	0.938	54.68±2.61	55.24±2.70	0.587	55.31±2.60	54.73±2.59	0.415
$CD56^{dim}CD16^{+}CD57^{+}$	$CD56^{dim}CD16^{+}$	58.10±2.32	57.80±1.97	0.792	57.20±2.13	57.37±2.15	0.494	62.26±2.77	62.43±2.75	0.853	62.83±2.80	61.85±2.71	0.494
$CD56^{dim}CD16^{-}CD57^{+}$	$CD56^{dim}CD16^{-}$	17.67±1.51	16.87±1.41	0.522	16.64±1.44	15.991.41	0.705	21.33±2.59	22.19±3.02	0.444	22.10±2.93	22.24±2.74	0.614

Supplementary Table 2A. NK cell activity before and after placebo treatment. Data are mean (±2SEM) for n= 21 and 28 subjects per group at four E/T ratios. Data were analysed using a paired samples t –test (before vaccination) and LMM (after vaccination). There were no statistically significant effects of the placebo treatment, either before or after vaccination.

Total NK cell activity

	**Young cohort**						**Older cohort**					
					**Before vaccine**	**After vaccine**					**Before vaccine**	**After vaccine**
**E/T ratio**	**baseline**	**week 4**	**week 6**	**week 8**	*P*- value Wk0vs4	*P*- value LMM	**baseline**	**week 4**	**week 6**	**week 8**	*P*- value Wk0vs4	*P*- value LMM
100:1	21.43±1.58	22.69±2.24	25.93±2.37	25.72±2.03	0.145	0.249	26.42±2.18	28.34±2.25	26.54±1.95	27.44±1.80	0.316	0.093
50:1	10.29±1.21	13.09±1.70	12.90±1.64	12.81±1.40	0.109	0.695	12.54±1.38	15.53±1.58	13.97±1.14	13.78±1.15	0.283	0.387
25:1	4.02±0.51	4.98±0.65	4.81±0.65	4.87±0.59	0.123	0.973	5.22±0.45	5.64±0.80	4.53±0.45	4.88±0.42	0.037	0.423
12.5:1	1.86±0.23	2.16±0.21	1.85±0.23	1.61±0.23	0.201	0.652	2.37±0.32	2.21±0.25	1.87±0.23	2.24±0.34	0.123	0.551

NK cell activity on per cell basis

	**Young cohort**						**Older cohort**					
					**Before vaccine**	**After vaccine**					**Before vaccine**	**After vaccine**
**E/T ratio**	**baseline**	**week 4**	**week 6**	**week 8**	*P*- value Wk0vs4	*P*- value LMM	**baseline**	**week 4**	**week 6**	**week 8**	*P*- value Wk0vs4	*P*- value LMM
100:1	1.62±0.24	1.84±0.29	2.18±0.40	2.43±0.39	0.173	0.657	1.30±0.15	1.38±0.17	1.38±0.15	1.28±0.15	0.209	0.868
50:1	0.78±0.15	0.96±0.17	1.13±0.24	1.21±0.19	0.061	0.761	0.63±0.08	0.76±0.10	0.72±0.08	0.65±0.08	0.524	0.778
25:1	0.33±0.07	0.34±0.06	0.38±0.07	0.43±0.06	0.463	0.690	0.25±0.03	0.29±0.07	0.24±0.03	0.22±0.02	0.919	0.608
12.5:1	0.14±0.03	0.15±0.02	0.17±0.04	0.14±0.03	0.652	0.977	0.11±0.02	0.10±0.01	0.09±0.01	0.10±0.01	0.795	0.725

Supplementary Table 2B. NK cell activity before and after the treatment with *B. longum* + Gl-OS. Data are mean (±2SEM) for n=32 and 25 subjects per group at four E/T ratios. Data were analysed using a paired samples t –test (before vaccination) and LMM (after vaccination). There were no statistically significant effects of treatment, either before or after vaccination.

Total NK cell activity

	**Young cohort**						**Older cohort**					
					**Before vaccine**	**After vaccine**					**Before vaccine**	**After vaccine**
**E/T ratio**	**baseline**	**week 4**	**week 6**	**week 8**	*P*- value Wk0vs4	*P*- value LMM	**baseline**	**week 4**	**week 6**	**week 8**	*P*- value Wk0vs4	*P*- value LMM
100:1	25.05±2.19	25.32±1.80	27.23±1.85	29.82±2.28	0.894	0.249	27.64±2.55	31.79±2.77	31.28±2.86	32.97±2.09	0.015	0.093
50:1	12.94±1.65	13.31±1.36	13.37±1.29	14.05±1.47	0.698	0.695	12.96±1.34	15.40±1.78	16.54±1.40	16.03±1.26	0.095	0.387
25:1	3.98±0.49	4.91±0.58	4.48±0.53	4.98±0.62	0.006	0.973	4.95±0.53	4.94±0.66	5.56±0.59	6.20±0.68	0.996	0.423
12.5:1	1.69±0.21	1.77±0.26	1.55±0.22	1.89±0.27	0.696	0.652	2.10±0.29	2.28±0.24	2.56±0.35	2.19±0.32	0.533	0.551

NK cell activity on per cell basis

	**Young cohort**						**Older cohort**					
					**Before vaccine**	**After vaccine**					**Before vaccine**	**After vaccine**
**E/T ratio**	**baseline**	**week 4**	**week 6**	**week 8**	*P*- value Wk0vs4	*P*- value LMM	**baseline**	**week 4**	**week 6**	**week 8**	*P*- value Wk0vs4	*P*- value LMM
100:1	1.97±0.28	2.29±0.36	2.22±0.33	2.54±0.42	0.183	0.657	1.22±0.16	1.33±0.18	1.41±0.17	1.42±0.16	0.203	0.868
50:1	1.04±0.19	1.19±0.20	1.03±0.12	1.19±0.21	0.275	0.761	0.58±0.08	0.63±0.11	0.76±0.09	0.68±0.08	0.497	0.778
25:1	0.33±0.05	0.44±0.08	0.35±0.05	0.42±0.08	0.021	0.690	0.21±0.03	0.19±0.03	0.25±0.03	0.25±0.03	0.606	0.608
12.5:1	0.14±0.03	0.15±0.03	0.11±0.02	0.17±0.03	0.585	0.977	0.09±0.01	0.09±0.01	0.11±0.02	0.09±0.01	0.850	0.725
